# Is Metabolic Rate Increased in Insomnia Disorder? A Systematic Review

**DOI:** 10.3389/fendo.2018.00374

**Published:** 2018-07-16

**Authors:** Julia L. Chapman, Maria Comas, Camilla M. Hoyos, Delwyn J. Bartlett, Ronald R. Grunstein, Christopher J. Gordon

**Affiliations:** ^1^CIRUS, Centre for Sleep and Chronobiology, Woolcock Institute of Medical Research, The University of Sydney, Sydney, NSW, Australia; ^2^Sydney Local Health District, Sydney, NSW, Australia; ^3^Faculty of Medicine and Health, Sydney Medical School, The University of Sydney, Sydney, NSW, Australia; ^4^Faculty of Science, School of Psychology, The University of Sydney, Sydney, NSW, Australia; ^5^Faculty of Medicine and Health, Susan Wakil School of Nursing and Midwifery, The University of Sydney, Sydney, NSW, Australia

**Keywords:** metabolic rate, insomnia, hyperarousal, sleep disturbances, systematic review

## Abstract

**Background:** Insomnia disorder is a highly prevalent health condition, affecting ~10–15% of the adult population worldwide. A central feature of insomnia is hyperarousal characterized as persistent and increased somatic, cognitive and cortical stimulation. Hyperarousal leads to a state of conditioned arousal that disrupts both sleep and daytime function. Research studies have shown increases in body temperature, heart rate, electroencephalographic activity, catecholamines, and oxygen consumption as a measure of metabolic rate. These findings provide evidence of increased physiological activation in insomnia however results are not consistent. The aim of the systematic review was to determine if metabolic rate in patients with insomnia is increased in keeping with the hyperarousal hypothesis.

**Methods:** We searched Pubmed, Web of Science, CINAHL, PsycINFO, EMBASE, and Scopus databases for observational and interventional studies that have measured metabolic rate in insomnia. Study characteristics were extracted and summarized and a risk of bias was performed for each of the studies.

**Results:** Two reviewers screened 963 abstracts with 35 articles of interest for full-text review. Four articles evaluating 75 participants were included in this systematic review. Two studies showed increased oxygen consumption across 24 h in insomnia patients compared with good-sleeping controls. One study which measured oxygen consumption at only a single timepoint showed no difference between insomnia patients and good-sleeping controls. A further study evaluating the effect of lorazepam on oxygen consumption in patients with chronic insomnia showed that lorazepam reduced metabolic rate during the night time only.

**Conclusions:** These findings show that metabolic rate appears to be increased across 24 h in line with the hyperarousal model of insomnia. However, these increases in metabolic rate in insomnia were minor compared to good-sleeping controls and the clinical significance is unclear. Larger, methodologically robust studies are required to confirm these findings and the effect of any increase in metabolic rate on sleep-wake disturbances or pathophysiology.

## Introduction

### Rationale

Insomnia disorder is a highly prevalent health condition, accounting for ~10–15% of the adult population globally (1). It is diagnosed using subjective symptomology consisting of difficulty initiating sleep, maintaining sleep or early morning awakenings, or a combination of these, with concomitant daytime impairments for at least 3 nights per week and 3 months duration despite adequate opportunity to sleep (2). The disorder is complex with considerable heterogeneity and has shown to be highly persistent, with longitudinal studies showing insomnia symptoms present over 1 year (3–5). Insomnia negatively effects quality of life, mood (anxiety and depression), cognitive performance, and daytime functioning (6–9), with these symptoms driving treatment-seeking behavior (10).

The pathophysiological mechanisms responsible for insomnia disorder have yet to be fully elucidated. Several different models have been proposed. Spielman's 3-P model is the most prominent, using a diathesis-stress model to describe how insomnia develops and is maintained over time through predisposing, precipitating, and perpetuating factors (11). Behavioral and cognitive models have also evolved (12, 13) which incorporate conditioning and dysfunctional beliefs which promote negative perceptions of sleep. More recently, a neurobiological model of sleep-wake dysregulation caused by regional-specific neural activity that promotes wakefulness during sleep has been proposed (14). This model unites psychological aspects of insomnia to neurobiological mechanisms.

One aspect that has underpinned these models is the presence of somatic arousal in insomnia patients. Evidence has demonstrated that insomnia is characterized by persistent and increased somatic, cognitive, and cortical stimulation (14). This has been termed *hyperarousal* which is thought to be present over 24 h (both sleep and awake) leading to chronic sleep disruption and impairments in daytime function. There are a number of research studies that have shown increased cognitive and physiological activation in insomnia patients compared to good-sleeping controls. In particular, patients with insomnia have shown an increase in body temperature, heart rate, cortisol and catecholamines (15). Collectively, these findings suggest that 24-h metabolic rate will be elevated in insomnia patients compared to good-sleeping controls. The sleep-wake cycle and basal metabolism are intrinsically linked with pronounced reduction in body temperature occurring at sleep onset and about 15% reduction in metabolic rate during sleep (16). An elevation in metabolic rate in insomnia may affect sleep leading to greater hyperarousal and perpetuating insomnia symptoms.

### Objectives and research question

We sought to determine whether metabolic rate is elevated in insomnia patients which would provide strong evidence of whole-body physiological hyperarousal. To date, there has been no systematic review of metabolic rate in insomnia patients. Therefore, the aim of the systematic review was to determine the metabolic rate of insomnia disorder patients.

## Methods

### Search strategy and data sources

An extensive and systematic search for studies on metabolic rate on insomnia populations was conducted using the following databases: PubMed, Scopus, Web of Science, CINAHL, Embase, and Psycinfo. The search terms selected were incorporated in the following Boolean expression: (“Metabolic rate” OR “Exercise capacity” OR “Energy metabolism” OR “Energy transfer” OR “Oxygen consumption” OR “Respiratory exchange ratio” OR “Oxygen utilization” OR “Energy utilization” OR “Energy expenditure” OR calorimetry) AND insomnia. The search terms were adapted when necessary to fit the specific search requirements of each database (see Supplementary Material). The primary search was undertaken on the 4th December 2017. No limitations were used in any database. After exporting articles into EndNote, duplicates were removed. Reference lists of relevant original and review articles identified through the search were searched for potential missed publications. The search was conducted using the Preferred Reporting Items for Systematic Reviews and Meta-analyses (PRISMA) statement and documented using the PRISMA flow chart (17).

Our primary study question was whether or not metabolic rate, determined using gold-standard measurement (calorimetry), was increased in patients with untreated insomnia disorder compared with good-sleeping controls or compared to patients with insomnia undergoing treatment.

### Study selection and data extraction

Articles were evaluated against the following inclusion criteria:

1. The article had to contain original data (i.e., was not a review or editorial).
2. Population: diagnosis of insomnia disorder, ≥18 years old, non-shift-working, non-jet-lagged, otherwise healthy.
3. Compared to good-sleeping controls or post-intervention.
4. The primary or secondary outcome had to be the measurement of metabolic rate directly (e.g., calorimetry).
5. Type of study could be either observational case-control study or interventional.

In a first phase, the studies were independently reviewed by two authors (JC, MC) using the title and the abstract. Disagreements in abstract inclusion or exclusion were resolved by consensus with a third author (CG). In a second phase, full text articles were independently reviewed by the same authors (JC, MC) and disagreements were resolved by consensus with the third author (CG). Full text articles were selected using the selection criteria and included in the final inclusion list. The characteristics, measurements and outcomes of the selected studies were extracted in duplicate (JC, MC) into a table template. Results were tabulated as mean ± *SD* where possible. Metabolic rate results were reported as $\overset{\cdot }{\text{V}}$O_2_ in ml/min. When these data were not available from an article, authors were contacted for the results or clarification.

Risk of bias was assessed for case-control studies using the National Institute for Health and Care Excellence methodology checklist (18). This checklist assisted with the assessment of the studies' internal validity by methodically appraising the selection of cases and controls, confounding factors and statistical methods. Risk of bias in interventional studies was evaluated using the Cochrane collaboration's tool for assessing risk of bias (19). This tool assisted with the assessment of the studies' internal validity and detection of selection, performance, detection, attrition, or reporting bias.

## Results

### Study selection and characteristics

**Figure 1:**
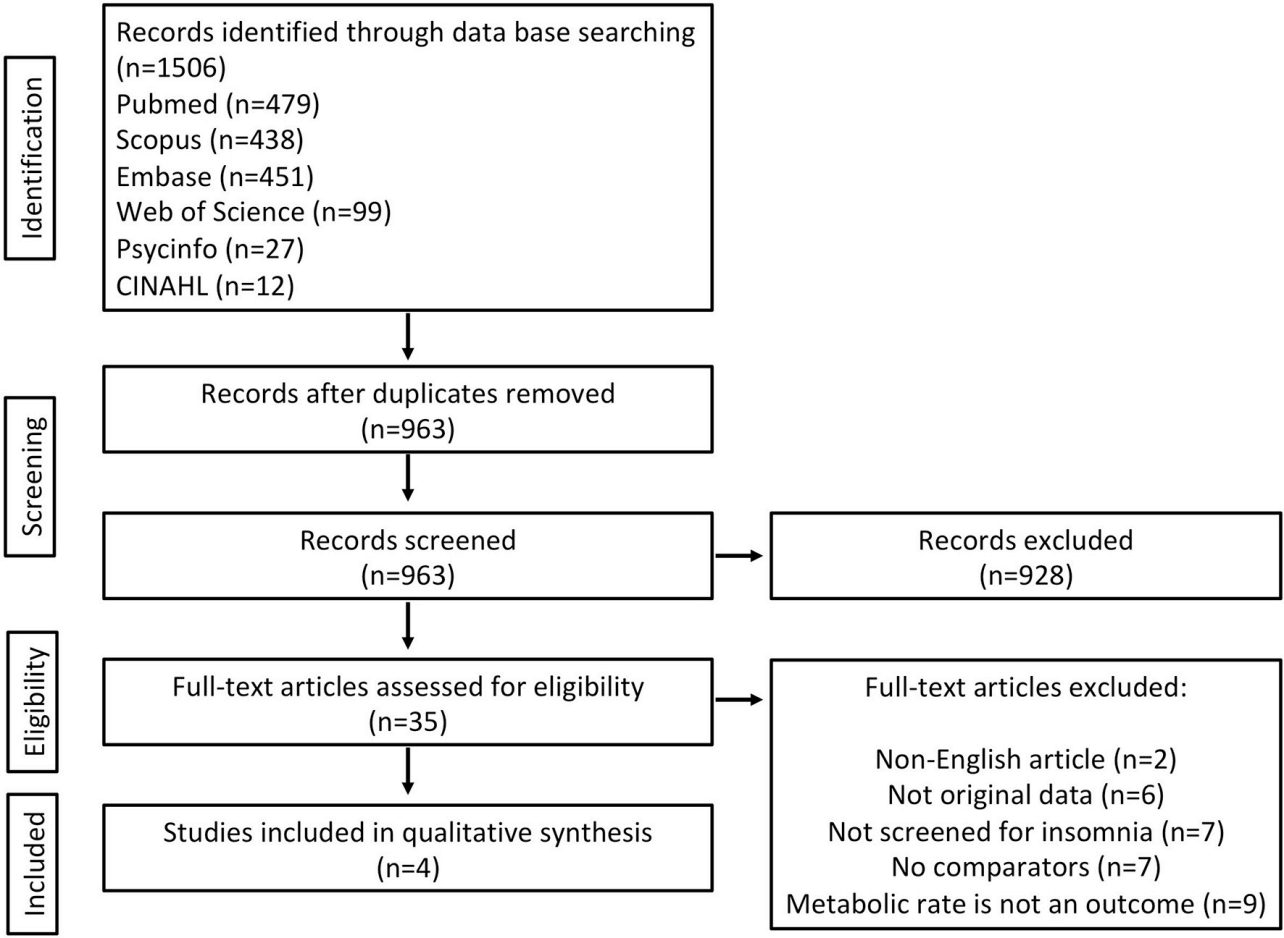
Flowchart of included studies (flowchart is modified from PRISMA) (17).

The primary search identified 1,506 records from six databases [Pubmed (*n* = 479), Scopus (*n* = 438), Embase (*n* = 451), Web of Science (*n* = 99), Psycinfo (*n* = 27), and CINAHL (*n* = 12)] (see Figure 1). After removing duplicates there were 963 records to screen for titles and abstracts initially. Following abstract screening 928 articles were excluded. The full-texts of the remaining 35 articles were checked for eligibility and of these, 31 were excluded (see Figure 1 for reasons). The four remaining articles (20–23) met the eligibility criteria and were included in this review, see Table 1. No further articles were identified through searching reference lists of reviews identified during the initial search nor the reference lists of the included articles. Authors were contacted directly when articles were missing data or information pertinent to the review (20–23). We received metabolic rate and some participant characteristic data (23). Unfortunately for the other studies (20–22) it was not possible to retrieve the standard deviations of waking metabolic rate measurements due to lack of access to the primary data. Due to a lack of information regarding variability within groups (e.g., standard deviations) from the four articles, we were unable to meta-analyse differences in metabolic rate between insomnia and good-sleeping controls.

**Table 1:** Characteristics of identified studies.

**References**	**Patient number, sex, age, BMI**	**Diagnostic criteria**	**Comparators number, sex, age, BMI**	**Case-match methodology**	**Sleep setting**	**Metabolic rate data capture**	**$\overset{\cdot }{\text{V}}$O_2_ RESULTS CASES ml/min**	**$\overset{\cdot }{\text{V}}$O_2_ RESULTS CONTROLS ml/min**
**CASE-CONTROL STUDIES**								
(20)	*n* = 10, sex unclear, Age 38.3 (7.1), selected for normal BMI	Screening questionnaire indicating sleep onset ≥45 min at least 4/7 OR were awake ≥60 min after falling asleep 4/7 and that this had existed for ≥year	*n* = 10, sex unclear (but matched with cases), Age 38.6 (6.8), selected for normal BMI	Indicated normal sleep on questionnaire with self-reported SOL <30 min and WASO <30 min. Matched to a case by sex, age (±5 years), weight (±25 lb), and general TIB characteristics	Sleep center referrals and ads to the local community. Urban Dept Veterans Affairs Medical Center, Dayton Ohio, USA	SensorMedics Deltatrac Metabolic Monitor using a mask and metabolic cart. Waking metabolic data recorded for 20 min immediately after awakening after one night and 20 min after 6 MSLTs during the day and 20 min prior to lights out. Sleeping metabolic data was measured throughout the entire 2nd night's sleep. VO2 automatically averaged at the end of each minute.	Overall: nr Wake: 296 (no SD) Sleep: 266 (no SD)	Overall: nr Wake: 266 (no SD) Sleep: 256 (no SD)
(21)	*n* = 9, 2 female, Age 31.7 (8.4), BMI 23.7 (3.3)	Screening questionnaire indicating sleep onset ≥45 min at least 4/7 OR were awake ≥60 min after falling asleep 4/7 and that this had existed for ≥1 year. Patients who demonstrated SOL <30 min and SE>90% and overestimated SOL by ≥100% on PSG and self-reported SOL≥20 min on both PSG nights were considered *sleep misperception insomniacs*	*n* = 9, 2 female, Age 32.8 (6.2), BMI 25.0 (3.4)	Indicated normal sleep on questionnaire with self-reported SOL <30 min and WASO <30 min. Matched to a case by sex, age, weight	Urban Dept Veterans Affairs Medical Center, Dayton Ohio, USA	As for (20)	Overall: 304 (26) Wake: 331(no SD) Sleep: 277 (no SD)	Overall: 286 (34) Wake: 266 (no SD) Sleep: 266 (no SD)
(23)	*n* = 13, all female, Age 51.7 (8), BMI 22.7(2.6)	Primary chronic insomnia using DSM-IV criteria	*n* = 12, all female, Age 52.8 (9.9), BMI 22.4(1.5)	Age and BMI matched good-sleeping controls	Depression and Sleep Research University, Psychiatric Uni Clinic Basel, Switzerland.	Indirect calorimetry (Deltatrac II, Datex) was conducted in the awake state (around 8 a.m.) for at least 20 min	Overall: n/a; Wake: 178.8(16.46); Sleep: n/a	Overall: n/a; Wake: 184.2(18.85); Sleep: n/a
**INTERVENTIONAL STUDY**							$\overset{\cdot }{\text{V}}$O_2_ **RESULTS Off drug**	$\overset{\cdot }{\text{V}}$O_2_ **RESULTS on lorazepam**
(22)	*n* = 12, 4 female, Age 36 (range 21–48), selected for normal BMI	Screening questionnaire indicating sleep onset ≥45 min at least 4/7 OR were awake ≥60 min after falling asleep 4/7 and that this had existed for ≥year	Crossover design. All participants received placebo and active medication.	Lorazepam 0.5 mg and 1.5 mg vs. no drug	Sleep center referrals or advertisements in local newspaper, Urban Dept Veterans Affairs Medical Center, Dayton Ohio, USA	As for Bonnet 1995	Overall: 334 (no SD) Wake: nr Sleep: 299 (no SD)	0.5mg Overall: 334 (no SD) Wake: nr Sleep: 285 (no SD) 1.5 mg Overall: 338 (no SD) Wake: nr Sleep: 287 (no SD)

*Data are mean (SD) unless other stated, BMI, Body Mass Index; DSM-IV, Diagnostic and Statistical Manual of Mental Disorders, 4th edition; SOL, sleep onset latency; WASO, wake after sleep onset; VO_2_, volume of oxygen consumption; n/a, data not collected; nr, not reported*.

### Synthesized findings

There were a combined total of 75 participants with and without insomnia disorder who underwent metabolic rate measurements in the four included articles. Three of the articles were case-control in design and used age, gender, and weight-matched good-sleeping controls (20, 21, 23). The other was a clinical trial assessing metabolic rate before and after treatment with two doses of lorazepam for insomnia (22). Participants in all studies were young to middle-aged (mean age ranged from 31 to 52 years), and were selected based on a healthy BMI. Three of the articles were published by the same research team (20–22) using the same indirect calorimetry technique to measure $\overset{\cdot }{\text{V}}$O_2_ (Deltatrac). This method utilized a metabolic mask worn by the participants during eight 20-min periods between waking and bedtime, and continuously overnight. The fourth study used a similar system (Deltatrac II) for measuring $\overset{\cdot }{\text{V}}$O_2_, but collected this only during a single 20-min period around 8 a.m. (23).

Metabolic rate was greater in the insomnia group compared with the good-sleeping control group in two of the case-control design studies (20, 21). This finding was consistent when measured during both the day and night. In the first study, all measurements across the 24-h collection period were elevated in insomnia patients and 9 out of 10 of these reached statistical significance (*p* < 0.01) (20). In their second study, they compared patients with sleep-state misperception insomnia/paradoxical insomnia (those who feel like they are awake throughout the night, but physiological measurement of their sleep deem them to be asleep) to good-sleeping controls. They reported overall that there was a statistically significant difference between the groups (*p* < 0.001) and a difference both separately during the day and night (*p* < 0.001 and *p* < 0.005, respectively, see Table 1 for mean differences) (21). In the case-control study by Seelig et al. (23) with all female participants only, $\overset{\cdot }{\text{V}}$O_2_ measured in the morning was marginally greater in the control group compared with the insomnia group but this was not statistically significant (See Table 1). In the clinical trial participants, overnight $\overset{\cdot }{\text{V}}$O_2_ was marginally decreased after taking lorazepam of either 0.5 or 1.5 mg dose (*p* < 0.02), but the daytime and 24-h values were the same in the no drug and lorazepam 0.5 and 1.5 mg conditions (22).

### Risk of bias

Overall, for the case-control studies, the risk of bias was evaluated as mixed (See Supplementary Material). All studies were shown to define clearly the case and control groups. However, two studies (20, 21) did not use a clinical diagnosis of insomnia disorder (for instance, DSM), but did report extensive questionnaire and diary data required for diagnosis of insomnia compared with good-sleeping controls (See Table 1). For the interventional study, there was no mention in the article regarding randomization, allocation concealment, or blinding, so it was unclear if this may have influenced the result (See Supplementary Material).

## Discussion

### Summary of main findings

The systematic review identified four studies that measured metabolic rate directly in patients with insomnia. Metabolic rate was found to be increased during both day and night in patients with untreated insomnia in the studies that sampled $\overset{\cdot }{\text{V}}$O_2_ across a 24 h period (20, 21). In contrast, when $\overset{\cdot }{\text{V}}$O_2_ was measured at only one morning timepoint (~8 a.m.), there was no difference between the insomnia and good-sleeping control groups (23). The final study compared $\overset{\cdot }{\text{V}}$O_2_ across the 24 h on and off lorazepam treatment for insomnia, showed that lorazepam reduced $\overset{\cdot }{\text{V}}$O_2_ during the night-time only (22). Overall these results indicate that metabolic rate across the 24-h period appears to be increased in insomnia patients when compared with age-, and gender-matched controls, which is consistent with hyperarousal model of insomnia. This result also aligns with recent findings that insomnia is associated with metabolic dysregulation compared with good-sleeping controls, suggesting that metabolic profiling may be a potential biomarker for disease risk in insomnia (24).

Methodological differences in the sampling period and study population, may account for observed $\overset{\cdot }{\text{V}}$O_2_ differences between the studies. Seelig et al. (23) measured $\overset{\cdot }{\text{V}}$O_2_ only during a single 20-min period shortly after waking, which may have been influenced by circadian variability, gender, and age-related differences from the studies by Bonnet and Arand (20–22). This study (23) only evaluated females who were on average about 15–20 years older than the participants in the other studies. Females on average have lower metabolic rates than men, and metabolic rate declines with age (25). It is unclear whether or not insomnia may affect metabolic rate differentially between males and females or across the lifespan. Additionally, the effect of benzodiazepines (lorazepam) on $\overset{\cdot }{\text{V}}$O_2_ may have been mediated by the alteration to neuroendocrine stress response (26). The nocturnal $\overset{\cdot }{\text{V}}$O_2_ was lower following administration of lorazepam, which may affect overnight cortisol secretion resulting in greater decrease in metabolic rate compared with the day. However, these results need to be interpreted with caution as the differences were marginal and the anxiolytic effects of lorazepam may have lowered overall $\overset{\cdot }{\text{V}}$O_2_ during the night (22). The increased $\overset{\cdot }{\text{V}}$O_2_ findings suggest that whole-body metabolic rate is elevated and supports the 24-h hyperarousal theory of insomnia (27). These data align with findings from a number of studies that have identified increased arousal in patients with insomnia across behavioral, cognitive, and autonomic nervous system domains (28). There is evidence of increased physiological activation in insomnia including heart rate, cortisol, body temperature, catecholamines, fast frequency electroencephaolography, and heart rate variability (15, 29). The findings of this review showing increased metabolic rate in insomnia aligns with this overall increased physiological activation. However, greater methodological rigor may be required to replicate and confirm the findings across these multiple physiological domains, as they are from small studies, and often are not repeated using similar methodologies enabling collation of data into meta-analyses (29).

It is possible that differences in metabolic rate between those with insomnia and good-sleeping controls could relate to different perceptions of a new environment, inducing different levels of a stress response (30). Sleep quality and quantity of people with insomnia may be affected by “first night effect,” where sleep is impaired due to sleeping in a new environment for the first time (31). Conversely, other people with insomnia will experience “reverse first night effect” where sleep is improved due to the new environment that is often devoid of stimuli that promotes insomnia symptoms (32). As the recordings were measured during the first night in the laboratory, it is possible that this may have influenced the difference between good-sleeping control and insomnia groups.

What is interesting to note, is that $\overset{\cdot }{\text{V}}$O_2_ measured using calorimetry during rest in these studies revealed different results compared with the broader literature using other measures of metabolic rate. A commonly used metabolic equivalent (METs) are calculated based upon diary data that derives a metabolic rate for different activities of daily living (e.g., time spent sitting, sleeping, moderately exercising) (33). One study has shown that METs were not increased in insomnia compared with good-sleeping controls (34). In particular, males with insomnia [*n* = 40, on average overweight, BMI 29.6(SD 3.5)], their METs were lower than both normal weight (*n* = 48) and overweight (*n* = 75) good-sleeping controls. This raises the question, are patients with insomnia equally active as controls but their metabolic rate at rest is elevated compared to controls? The decreased activities of daily living could be a result of the behavioral components of insomnia, whereby patients feel less able to exercise or spend more time lying down as a result of their condition.

In two other studies, maximum oxygen uptake ($\overset{\cdot }{\text{V}}$O_2_ peak) was used to examine exercise capacity, comparing those with insomnia symptoms from questionnaire and those without (34, 35). In both studies, $\overset{\cdot }{\text{V}}$O_2_ peak was lower in those with insomnia symptoms, even after adjusting for age, sex, and other potential confounders. This shows that people with insomnia symptoms appear to have decreased exercise capacity. These results need to be explored in an otherwise healthy clinical insomnia population, but they suggest that maximum oxygen capacity may be lower in insomnia disorder compared to good-sleeping controls. In our review resting metabolic rate was elevated in insomnia compared to controls. Further research is required to determine if elevated resting metabolic rate coexists with decreased exercise capacity in diagnosed insomnia disorder and how this relates to disease risk profile.

## Limitations

A major limitation of this review is that of the four articles identified in the final search, three were from the same research group within a single sleep center, suggesting their findings may not be representative of insomnia. As the only study completed by a different research team (23) showed no difference in metabolic rate between insomnia and good-sleeping control groups, any conclusions pointing toward higher metabolic rate in insomniacs comes from a single research team. Further studies from different teams, with a larger number of participants, taking into account the potential for first night effects would be required to confirm this finding. All of the studies had small to modest sample sizes, resulting in only 32 patients with insomnia disorder to be compared with 31 good-sleeping controls and only 12 participants who were measured before and after treatment with lorazepam. The clinical trial had high risk of bias due to unclear methods regarding randomization and blinding, and placebo effects are known to be strong in insomnia, potentially affecting the validity of the result (36). The participants in these studies were selected for healthy weight and were on average middle-aged. The results of these studies could therefore not be extrapolated to children or older adults, or those who are overweight. Data was also unable to be meta-analyzed as we were unable to retrieve all standard deviations. We believe the measurement of oxygen consumption in a whole room calorimeter may solve a lot of the methodological problems (37).

## Conclusions

The results from a small number of studies suggest that metabolic rate appears to be increased in patients with insomnia disorder across 24-h in line with the hyperarousal model of insomnia. These findings need to be replicated in larger prospective studies. Clinical trials evaluating the effect of insomnia therapies, such as cognitive behavioral therapy, on metabolic rate would be useful in determining causality.

## Author contributions

CG, JC, and MC contributed to the conception and design of the systematic review. JC and MC independently reviewed abstracts and papers and disagreements were resolved by consensus with CG. CH did the risk of bias. CG, JC, and MC wrote sections of the manuscript. DB and RG revised the manuscript and contributed with intellectual ideas. All authors contributed to manuscript revision, read, and approved the submitted version.

### Conflict of interest statement

The authors declare that the research was conducted in the absence of any commercial or financial relationships that could be construed as a potential conflict of interest. The reviewer KD and handling Editor declared their shared affiliation.
