# Supplementary material for: Is Metabolic Rate Increased in Insomnia Disorder? A Systematic Review
Source: Front Endocrinol (Lausanne). 2018 Jul 16;9:374. doi: 10.3389/fendo.2018.00374 (PMC6054926; doi:10.3389/fendo.2018.00374)
Supplement: Supplementary file 1 [file Data_Sheet_1.docx]

# Supplementary Material

## Search methodology

Boolean expressions adapted to each database are shown below:

Pubmed. Date of search: 4/12/2017. It yielded 479 abstracts. No limits applied.

(("Metabolic rate" [Title/Abstract] OR "Exercise capacity" [Title/Abstract] OR "Energy metabolism" [Title/Abstract] OR "Energy transfer" [Title/Abstract] OR "Oxygen consumption" [Title/Abstract] OR "Respiratory exchange ratio" [Title/Abstract] OR "Oxygen utilisation" [Title/Abstract] OR "Energy utilisation" [Title/Abstract] OR "Energy expenditure" [Title/Abstract] OR calorimetry [Title/Abstract] AND insomnia OR insomniac [Title/Abstract])) OR ("Metabolic rate" OR "Exercise capacity" OR "Energy metabolism" OR "Energy transfer" OR "Oxygen consumption" OR "Respiratory exchange ratio" OR "Oxygen utilisation" OR "Energy utilisation" OR "Energy expenditure" OR calorimetry AND insomnia OR insomniac)[MeSH Terms].

Scopus. Date of search: 4/12/2017. It yielded 438 abstracts. No limits applied.

TITLE-ABS-KEY (("Metabolic rate" OR "Exercise capacity" OR "Energy metabolism" OR "Energy transfer" OR "Oxygen consumption" OR "Respiratory exchange ratio" OR "Oxygen utilisation" OR "Energy utilisation" OR "Energy expenditure" OR calorimetry AND insomni*))

Web of science. Date of search: 4/12/2017. It yielded 99 abstracts. No limits applied.

TS= ("Metabolic rate" OR "Exercise capacity" OR "Energy metabolism" OR "Energy transfer" OR "Oxygen consumption" OR "Respiratory exchange ratio" OR "Oxygen utilisation" OR "Energy utilisation" OR "Energy expenditure" OR calorimetry) AND insomni*

OR

TI=( "Metabolic rate" OR "Exercise capacity" OR "Energy metabolism" OR "Energy transfer" OR "Oxygen consumption" OR "Respiratory exchange ratio" OR "Oxygen utilisation" OR "Energy utilisation" OR "Energy expenditure" OR calorimetry ) AND insomni*

CINAHL. Date of search: 4/12/2017. It yielded 12 abstracts. No limits applied.

# Query Limiters/Expanders

S8 S6 AND S7 Search modes - Boolean/Phrase

S7 (MH "Insomnia") OR "insomni*" Search modes - Boolean/Phrase

S6 S1 OR S2 OR S3 OR S4 OR S5 Search modes - Boolean/Phrase

S5 (MH "Calorimetry") OR "calorimetry" Search modes - Boolean/Phrase

S4 (MH "Oxygen Consumption+") OR "Oxygen Consumption" Search modes - Boolean/Phrase

S3 (MH "Energy Transfer") OR "Energy Transfer" Search modes - Boolean/Phrase

Embase. Date of search: 4/12/2017. It yielded 451 abstracts. No limits applied.

(("Metabolic rate" or "Exercise capacity" or "Energy metabolism" or "Energy transfer" or "Oxygen consumption" or "Respiratory exchange ratio" or "Oxygen utilisation" or "Energy utilisation" or "Energy expenditure" or calorimetry) and insomni*).mp. [mp=title, abstract, heading word, drug trade name, original title, device manufacturer, drug manufacturer, device trade name, keyword, floating subheading word]

Psycinfo. Date of search: 4/12/2017. It yielded 27 abstracts. No limits applied.

"Energy metabolism" or "Energy transfer" or "Oxygen consumption" or "Respiratory exchange ratio" or "Oxygen utilisation" or "Energy utilisation" or "Energy expenditure" or calorimetry) and insomni*).mp. [mp=title, abstract, heading word, table of contents, key concepts, original title, tests & measures]
