# Supplementary figures and images for: Is Metabolic Rate Increased in Insomnia Disorder? A Systematic Review
Source: Front Endocrinol (Lausanne). 2018 Jul 16;9:374. doi: 10.3389/fendo.2018.00374 (PMC6054926; doi:10.3389/fendo.2018.00374)

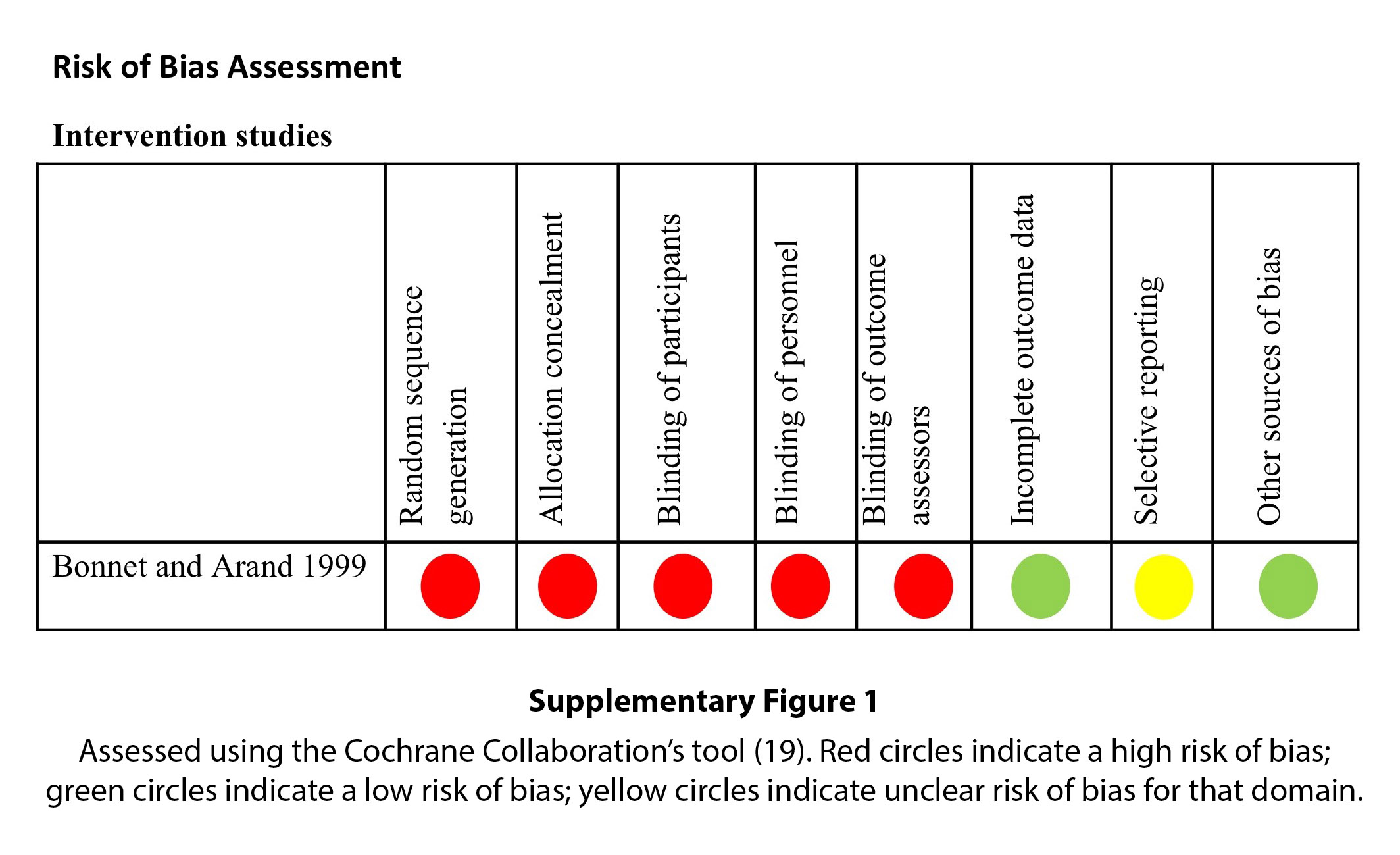

Supplement: Supplementary file 2 [file Image_1.jpg]

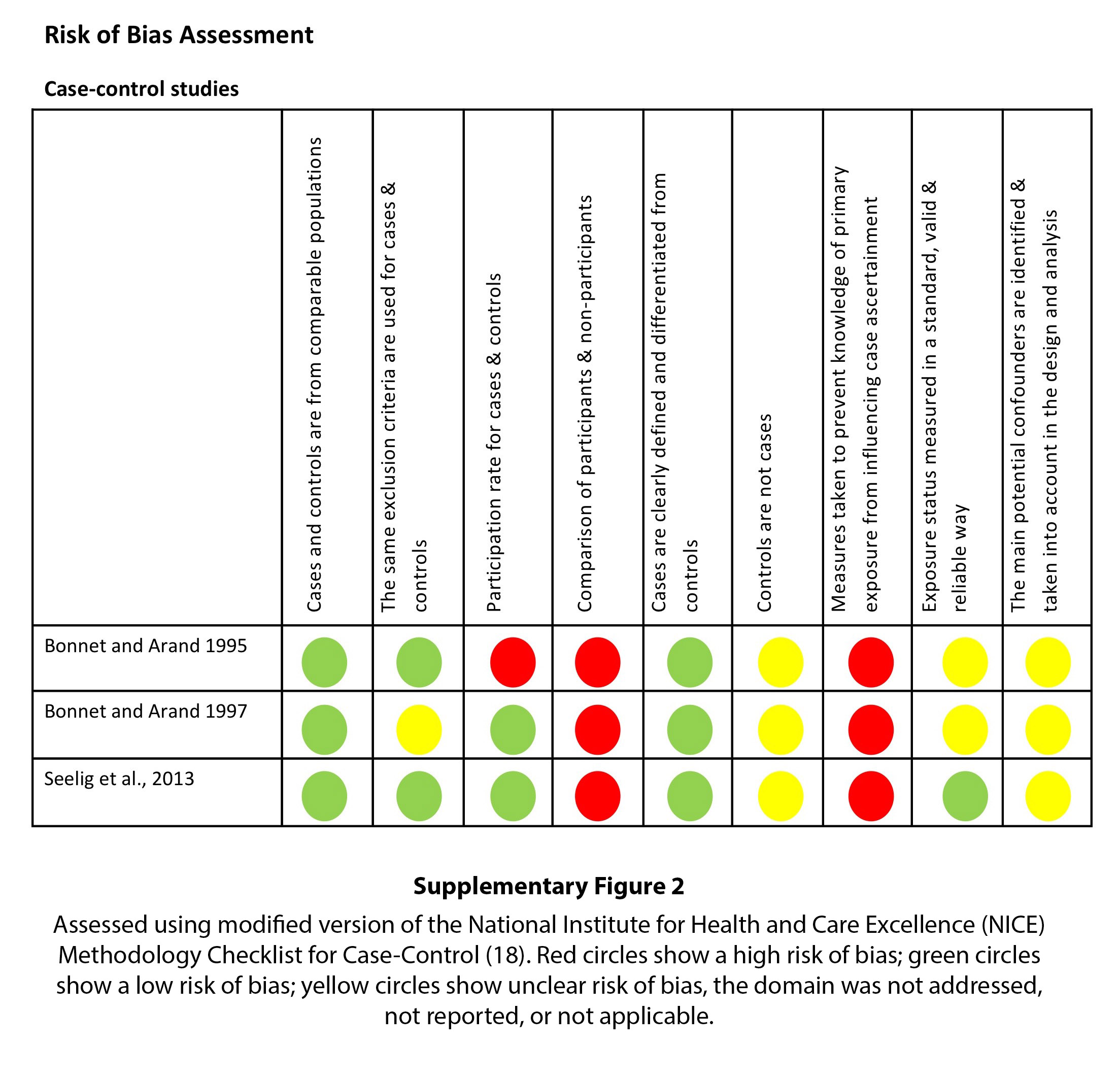

Supplement: Supplementary file 3 [file Image_2.jpg]
